# Lessons from the COVID pandemic in music education the advantages and disadvantages of online music education

**DOI:** 10.1016/j.heliyon.2024.e35357

**Published:** 2024-08-02

**Authors:** Judit Váradi, J. Miklós Radócz, Ádám Mike, Zoltán Óváry, Gabriella Józsa

**Affiliations:** a Music Faculty, University of Debrecen, 4032, Debrecen, Nagyerdei krt. 82, Hungary; b Doctoral School of Human Sciences, University of Debrecen, 4032, Debrecen, Egyetem tér 1, Hungary; c Doctoral School of Human Sciences, University of Debrecen, Faculty of Music University of Debrecen, 4032, Debrecen, Nagyerdei krt. 82, Hungary; d Károli Gáspár University of Reformed Church, Faculty of Pedagogy, 2750 Nagykőrös, Hősök tere 5, Hungary

**Keywords:** Music education, Online education, COVID-19, Innovation of music education, Music teacher

## Abstract

The COVID-19 pandemic had far-reaching effects on various areas of everyday life. In several countries, singing, the most accessible form of musical expression, was banned, as was the use and teaching of wind instruments. Nevertheless, innovations in music education combined with teachers' dedication and creativity introduced several elements into the teaching process which are worth using in the future.

The aim of our study is to explore the experiences of this novel form of music education among teachers at the primary, secondary, and tertiary levels in Hungary and abroad. The respondents in our survey answered open-ended questions on the advantages and disadvantages they had experienced with online education. The MAXQDA software was used for the qualitative analysis of the data collected. Music teachers highlighted the disadvantages of online education. As for positive responses, teachers mentioned the improvement of digital competencies and the development of students' autonomy.

## 1 Introduction

The continuous development of Information and Communication Technology (ICT) for the 21st century has a significant impact on all aspects of life. The need to effectively integrate ICT tools into education has been an ongoing effort for many years. The emergence of mobile technology in everyday life, tablets, mobile phones, represents a new form of connectivity and collaboration, which also represents an opportunity for innovation in education. However, improving digital infrastructure and making tools available does not automatically lead to a digital switchover in education. Even though educational innovation and softver development have continued to progress, music education has not changed significantly in recent decades. Despite the experimental presence of digital technology in music subjects in public education, classical instrumental education has stuck to tradition. The introduction of online education in an emergency situation has also caught those involved in music education unprepared and unaware. Summing up the advantages and disadvantages of digital distance learning in music education, the question arises how the “good practices”, creative ideas, teacher motivation strategies to help students' activity, valuable online resources developed over the past months can be adapted to a modern art education system. This paper collects the experiences of this period, based on the opinions of music teachers in Hungary.

**Fig. 1:**
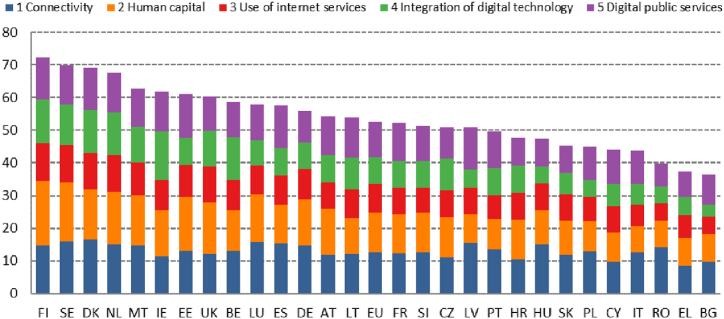
Digital economy and Society Index, 2020. Source: Desi 2020.

Different countries use different indicators to assess the impact of the digital economy on sustainable development.There are many different measures of the digital economy, such as the Network Readiness Index (NRI), and Digital Acceptance Index (DAI) [1], but the best known and most commonly used is the Digital Economy and Society Index (DESI), which has been used by many researchers [2], and we have chosen it to explore the technical background of online education. To explore the technical background of online education, we used the five-dimensional report of the Digital Economy and Society Index [3], which provides the most detailed data for Hungary. The examined indicators included (1) connectivity, measured by the coverage of fixed and mobile broadband Internet; (2) human capital, measured by individuals’ Internet use and the share of ICT specialists, which covered both basic and advanced digital skills; (3) the use of Internet services, such as access to digital transactions, online content, and online communication; (4) the integration of digital technology, including the use of social media, the digitalisation of businesses, and e-commerce; and finally (5) digital public services, including fully-online administration, e-government, and e-health. Finland, Sweden, Denmark, and the Netherlands were the most advanced, with Hungary ranked 21st (Fig. 1). The results are interesting because they already include the consequences of the digital transformations due to the pandemic. The *human capital* dimension shows that more than half of the population lacks basic digital skills. The *use of internet services* displays an improving trend, with a significant increase in digital banking (58 %) and online shopping (59 %), but the results are still below the EU average. Hungary lags significantly behind the EU average in two areas. One is the *integration of digital technologies* and the use of ICT tools in businesses, where Hungary ranks 26th. The other field where significant improvement is needed relates to *digital public services*, with the country ranking 24th [4].

According to the European Joint Research Centre of the European Commission [5], in 21 European countries, a fifth of children do not have access to the Internet, so the computer at home is not suitable for learning, while a quarter of children do not have their own room or a similar quiet environment when their family is at home. The digital gap, or digital inequality, reveals differences between groups in the generational, global, geographical, or social sense [6].

According to the McKinsey report [7] on 25 education systems around the world, the teacher is pivotal to the best performing systems regardless of culture, so to raise the quality of education, the right person needs to be equipped with effective tools to become a teacher and provide the best possible education for all children. The role of teachers has become even more important during the unexpected situation of COVID-19 and lockdowns.

Music teachers had to find solutions for teaching during COVID-19 under different circumstances across countries. Diverging regulations were in place not only across countries but even within a country. For example, in Germany, the individual states and schools decided on the form of teaching. Kivi and colleagues [8] divide online education into five different phases, which can also be interpreted in an international context.

● Phase 1: No teaching: schools were closed so unexpectedly that online teaching did not start immediately as the education system had not prepared for this.
● Phase 2: Establishing contact by e-mail, messages, or even letters: this method provided children with many tasks, such as listening to certain pieces of music, reading various texts, researching information. Creative tasks were still rare at this stage. In this asynchronous form of learning and teaching, teachers and children did not meet directly and simultaneously in an online environment.
● Phase 3: Regular contact: after a few weeks, everyone had reached the stage where the different platforms were used regularly for communication.
● Phase 4: Hybrid teaching: after the school opened, online teaching was combined with in-person teaching.
● Phase 5: Return to face-to-face teaching: even at this stage, teachers faced major challenges due to central regulations, such as restrictions on singing, the use of masks, and mandatory distance.

During the COVID-19 pandemic, education was supported by educational programmes on radio and television. In addition to remote learning, home-based learning was also a common option, which left parents, guardians, and caregivers with the responsibility of teaching [9]. Young children are the most vulnerable, as they are more dependent on parental support. They are less able to adapt in the absence of human resources at home, such as older siblings [10].

## 2 The international experience of online music education during COVID-19

In addition to the practical implementation, researchers in the field of online music education have also looked at the technical solutions and the role of music education in this specific life situation. The mental health implications of the COVID-19 pandemic are far-reaching. Homeschooling alone can be a source of stress for families and students. In Thailand, a joint study by the United Nations and the Children and Youth Council of Thailand surveyed 6771 children. The survey found that more than seven in ten young people responded that the pandemic was affecting their mental health, causing stress, worry and anxiety [11]. Vega's study looked at the importance of music education during the pandemic. Music education helps people cope with the negative consequences of isolation, such as stress and anxiety. It also contributes to the harmonious development of children, i.e. it has a positive impact on the intellectual and emotional growth of the individual, which is particularly important in the current situation [12].

In a qualitative study, De Bruin (2021) investigated the teaching practices used by instrumental music teachers in secondary schools in Melbourne. A total of 15 teachers participated in the survey, the main aim of which was to explore perspectives on music teaching in the era of online distance learning. Thematic analysis of the interview transcripts revealed music teaching approaches that positively impacted students' empathy and supported their receptiveness to connectivity. Unconventional pedagogical practices reinforced and fostered interpersonal connection through musical experiences and explorations [13]. The interview survey by Biasutti, Antonini and Schiavio [14] aimed to investigate the teaching practices and strategies developed by music teachers during the pandemic emergency. A total of 15 instrumental and theory music teachers teaching in secondary schools from Europe and the United States participated in the study. The semi-structured interviews covered curriculum, lesson planning, assessment, examinations and timetabling. Participants were also asked about the advantages and limitations of digital teaching in addition to online teaching methods. Participants also made significant improvements in the use of skills such as flexibility, problem solving and creativity in curriculum planning and the use of various distance learning tools. They reported that online teaching was very time-consuming - for example, planning activities, preparing materials and exploring new possibilities for technical tools - and that it was demanding to find a work-life balance.

According to the summary of international research [15], the most striking disadvantages included the restricted feasibility of practical activities, the unsuitability of digital platforms for music activities in groups (choirs, orchestras, big bands, etc.), and the ban on live music performances. The material covered had to be reduced compared to the curriculum. The lack of live interaction meant that children's social skills could not develop. Inadequate ICT tools for teaching and slow or missing Internet connection also hindered education. Technological gaps also negatively affected learning, thus reinforcing social inequalities. Teachers' different, sometimes even insufficient, levels of digital competences and the lack of online education programmes also compromised the success of online education [15].

Findings from Portugal and Germany [16] show that teachers’ opinions on their experience of online education were almost unanimous. Among the benefits, teachers highlighted the technival area, such as use of audio or video recordings as a very useful method for both children and teachers, as children practiced more to be able to produce a better recording.

As teachers were unable to perform the works live and possibly find a performance of sufficient quality, teachers were also motivated to practice in order to help children with their own recordings. Thus, it can be said that online education in this area has contributed to the professional development of both the student and the teacher.

Another positive innovation was the abundance of live or recorded online concerts, which were sent to families or published on the school website, thus reaching a wider audience.

Among the new methodological elements, playlists compiled by music teachers, which children could listen to and thus expand their repertoire, proved useful.

Teachers' search for the best technical solution enhanced digital competence, which led to a better knowledge of resources, the development of digital skills, and the refinement of their own method of using the available tools in a virtuoso way. Through online education, the family became more involved in music education and better supported children on their journey. Another positive impact was that most schools provided an institutional e-mail address to children, so the student could be reached more easily. The researchers' findings reveal another benefit in terms of increased communication between colleagues. To reduce teachers’ isolation, institutional meetings and discussions in the online space became popular [16].

Johnson in an earlier study of experimental online music education in US universities, found that the most significant difficulties in distance learning arise in academic areas associated with acquiring practical skills [17].

According to a survey in China, students studying choral art generally positively received the introduction of distance learning. Most participants noted the saving of time they would have spent on the road to the educational institution as the advantage of this education format. Meanwhile, the practical part of the training is conducted in face-to-face classes, with the second semester of distance learning being devoted to theoretical subjects [18]. It can be stated that it is good to study the history and theory of music remotely, comparing the distance learning format with the in-person one. However it is somewhat problematic to teach practical skills [19].

Martínez also concluded that online education offers many advantages in terms of time and money savings, ecological issues, access from remote areas and interaction between students and teachers worldwide. A survey of conservatories and music schools in Spain found that most teachers preferred face-to-face lessons both before and after forced distance learning. Internet platforms are not currently suitable for creating synchronicity [[20], [21]]. Although face-to-face lessons cannot be fully substituted to guarantee quality education, online lessons can be a useful complement in certain situations [22]. Shaw and Mayo suggest that the following activities justify the expediency of introducing online learning technologies into music education: conducting training seminars, discussions on forums, completing test tasks [23].

## 3 Aim, design, and methodology of the study

In November 2020, the Art Education Research Group investigated the experiences and impact of online art education at all levels of education in Hungary, as well as at Hungarian-language educational institutions abroad and among Hungarian music teachers who taught outside Hungary, with the support of the Hungarian Academy of Arts Research Institute of Art Theory and Methodology.The study was conducted according to the guidelines of the Declaration of Helsinki, and approved by the Institutional Review Board of Doctoral Program on Educational Sciences, University of Debrecen, Hungary (5/2020). The aim of research is to explore the experiences the distance learning of music among teachers at the primary, secondary, and tertiary levels. The respondents in our survey answered open-ended questions on the advantages and disadvantages they had experienced with online education.

We chose convenience sampling for data collection [23]. Accordingly, a link to the survey was sent to the institutions via email and was also published on social media. Of the respondents of the survey, 13 % lived abroad, with Romania and Ukraine as the largest countries of residence after Hungary, but there were also responses from Austria, Switzerland, Slovakia, Italy, Germany, Finland, Estonia, and Canada.

The measurement tool for the study was a self-developed online questionnaire with 48 closed and open-ended questions for teachers of music education at the primary, secondary, and tertiary levels. The responses were aggregated into the Online Music Education (OME) database, which was the basis for our empirical analysis. To answer our research questions, we processed the responses of music teachers (music teachers in public education, teachers of solfeggio and music theory, instrumental teachers, and private singing instructors), accompanists, choir and orchestra conductors at the primary, secondary, and tertiary level using. A total of 352 respondents completed the questionnaire, with the over-representation of women (73 %). The average age of music teachers who completed the questionnaire was 44 years; the youngest respondent was 21, and the oldest was 70.

We examined the proportion of positive and negative responses in relation to attitudes towards online education based on the experiences of the music teachers in the sample. We considered the respondents’ views on the lack of personal presence, their adaptation to the digital environment, and their opinion on the most positive aspects of online education.

To objectively analyse the open-ended responses, we used the MAXQDA qualitative data analysis software, which allowed us to quantify the written responses, to quantitatively analyse coherent text fragments, and to organise information in a transparent way. The advantage of the software is that possible subjective elements can be avoided. In the content analysis, we followed a three-stage method [24]. In the first stage, we coded from the responses the words and text fragments with evaluable meaning. In the second stage, we analyzed the frequency of the codes, and generated subcodes accordingly. In the third stage, we interpreted the results and the relationships that emerged.

The coding followed a deductive method in that the main codes were generated based on the hypotheses. The content analysis, however, required the use of the inductive method, which prompted additional main codes [25,26]. The eight main codes were then ranked and further divided into subcodes. Several difficulties were encountered in the coding process. Categorisation was problematic when a response could feasibly belong to more than one main code or subcode; for example, it was not always immediately obvious whether the reported “*poor quality*” related to a problem with the equipment, network, or instrumental performance. Moreover, there were several conflicting answers to the original questions. This means that the disadvantages were accompanied by some positive experiences, while the advantages prompted several negative responses. We illustrated the coding process by the following example. One respondent's opinion on the impact was: “Impersonal, difficult to hold accountable, lack of motivation.” The impersonal indicator was categorised under the main code related to Relationship and communication, Emotional factors sub-code. Difficulties with accountability were grouped under the main code Checking, correcting, accountability. Lack of motivation was assigned to the main subcategory Mental and physical factors, under the subcategory Mental factors.

As a result of the coding, the overall number of occurrences of codes was 591 for advantages and 1111 for disadvantages. The difference between the two figures is noteworthy, especially the striking predominance of negative experiences. It is interesting to observe that the largest category for advantages was also the main category for disadvantages listed by respondents as advantages, see the value indicated in the bottom row of the table with 118 such cases (Table 1.).

**Table 1:** Rank and frequency of main codes and subcodes.

Codes, subcodes	Disadvantages rank	*Disadvantages frequency of codes*	Advantages rank	*Advantages frequency of codes*
Factors related to learning, teaching, and curriculum	1	*334*	4	*86*
Individual technical problems	1	*124*	–	*-*
Musical group activities	2	*85*	5	*1*
Methodology, teaching classes	3	*52*	2	*18*
Monitoring, correction, testing	4	*48*	3	*9*
Skill development	6	*10*	4	*8*
Educating for independence	–	*-*	1	*50*
Other	5	*15*	–	*-*
Infrastructural and digital factors	2	*312*	3	*89*
Managing digital tools and applications	1	*162*	–	*-*
Network problems	2	*129*	–	*-*
Instrument	3	*16*	4	*2*
Digital competences	4	*5*	1	*48*
Information storage, recording	–	*-*	2	*31*
Other	–	*-*	3	*8*
Factors related to contact and communication	3	*187*	1	*115*
Emotional factors	1	*143*	5	*5*
Factors related to teaching classes	2	*22*	1	*56*
Quality, success	3	*14*	4	*12*
Parental involvement	4	*8*	2	*27*
Familiarity with home environment	–	*-*	3	*15*
Factors related to success	4	*175*	6	*37*
Overall experience	1	*106*	2	*12*
Rate of progress	2	*39*	3	*6*
Cancelled live events	3	*17*	–	*-*
Impact on students	4	*13*	1	*19*
Factors related to mental and physical wellbeing	5	*69*	5	*36*
Mental factors	1	*53*	1	*36*
Physical factors	2	*16*	–	*-*
Spatial and temporal factors	5	*24*	2	*105*
Temporal factors	1	*18*	1	*68*
Spatial factors	2	*5*	3	*12*
Temporal and spatial factors	3	*1*	2	*25*
Factors related to health protection	–	*-*	7	*9*
**Response provided for the opposite category, not the right place**	**-**	**10**	**-**	**118**

## 4 Results and interpretation

The analysis and interpretation of the data are presented in descending order of the ranking of the main codes.

In the category of factors related to learning, teaching, and curriculum a very high number of disadvantages were identified (334 codes), while the number of advantages is negligible in comparison (86 codes). In categorising the disadvantages and advantages, four shared subcodes were created, with responses in relation to musical group activities, considerations of methodology and teaching, skill development, and finally problems of monitoring, correction, and testing. As a shared characteristic, all four subcodes in this main code were connected with more disadvantages than advantages.

When describing difficulties with *musical group activities*, respondents mentioned the impossibility of activities related to polyphonic singing, as “rhythmic exercises are not possible to perform together with students; there are no chamber music practice sessions, no choir and orchestra rehearsals, and no shared musical experiences”.

The practices related to *methodology and teaching classes* were characterised by ambiguous and difficult communication. Students found it more difficult to understand the material, and teaching became more complicated by the missing opportunities for immediate correction and improvement. Besides negative experiences, relevant positive aspects were also mentioned, including the need to rethink existing methods, the use of more varied exercises, and the effectiveness of music theory lessons.

The negative aspects of the *monitoring, correction, and testing* subcode related to the impaired ability of monitoring students, the difficulty of correction (both for technical problems and in writing), and the lack of oral tests. Positive opinions emphasised the increased opportunities for testing and easier assessment.

As for *skill development*, respondents considered the development of creativity, verbal skills, and adaptability to be effective online, but the improvement of certain musical skills and individual performance skills was not viewed as feasible.

In addition to the shared subcodes, we also created exclusively positive or negative subcodes. Respondents found the opportunity to increase students’ autonomy as a positive impact of online music education. Students learned to use the tuner, their self-confidence increased, and their self-reflection improved. As highlighted by some respondents, this only worked for older students. As for negative aspects, the most frequently mentioned disadvantage related to *individual technical problems* (124 codes). In online sessions, teachers could not correct hand, mouth, and body posture, were not able to improve intonation and sound, and were hindered in teaching and demonstrating sensitive musical elements and appropriate posture. Difficulties that could not be assigned to any other subcode were grouped into a separate category (15 codes). These included but were not limited to the problems of preparing for entrance examinations, hearing-based mistakes, and the lack of live music.

***Infrastructural and digital factors*** were of particular importance in the days of online education. The subcodes of advantages (89 codes) and disadvantages (312 codes) showed the most significant difference in this category. The only shared subcategory concerned *digital competences*. Teachers encountered substantial difficulties with using different platforms, having inadequate knowledge of technical tools, and the ability of students to intervene in the lesson. Positive aspects included the development of digital skills (both for teachers and students), the acquired familiarity with useful and creative applications, and the speed of access to information. In addition to the subcategory on digital competences, the usefulness of recordings and the possibility of storing information were also among the positive responses. Music teachers considered that listening to self-made recordings helped detect mistakes. In addition, the accessibility of the recorded material was assessed to be helpful in subsequent practice.

Three subcategories were created for negative opinions: *managing with digital tools and applications*, *network problems*, and problems with *instruments*. For the first subcode, in addition to the obvious IT issues and equipment-related negative experiences, respondents mentioned specific problems such as unbalanced dynamics, unsatisfactory quality for music, and the lack of real-time audio transmission.

Respondents identified the Internet connection and signal strength as major sources of problems. In addition to a lagging, poor-quality connection, there were several occasions when the application did not respond or quit on its own, the picture froze, and the sound disappeared.

There was a consensus that signal strength was inadequate in most institutions and homes. Nevertheless, the most annoying problem in relation to the quality of the network connection was the asynchronous transmission of picture and sound.

Respondents also had important observations on instruments. These included the issue of instrument maintenance and the impossibility of teaching if a student did not have an instrument at home or had only an inappropriate one.

Overall, a significant proportion of music teachers believed that the current state of technology was not sufficient for online music education.

***Factors related to contact and communication*** were included in the third main code. Two broad categories were distinguished: psychological or emotional factors, and physical factors, which directly related to teaching and contact with parents. Undoubtedly, the pandemic had a major impact on everyone involved in education, with the absence of personal relationships as a considerable psychological difficulty. Consequently, the most frequent disadvantages (187 codes) concerned *emotional factors* (143 codes), with the lack of personal presence and contact mentioned most often (101 codes). Other emotional disadvantages included the lack of community, impersonality, the weakening of social ties, and the deterioration of teacher-student relationships. It is interesting to note that most respondents mentioned as a positive aspect that the relationship between teachers and students became closer and deeper. When asked about the *factors related to teaching*, respondents mainly mentioned practical benefits such as continuity, easy access to students, reduced absences, and the fact that lessons could be held even in the case of illness. Negative experiences related mainly to the lack of feedback and immediate reaction. On both sides, the role of parents was also mentioned. Parental support was deemed essential, especially for young children and beginners, but the results in terms of implementation were varied.

In the assessment of the quality and success of contact and communication, many music teachers found the new ways of contact diverse and effective. However, there were some who found these problematic and linked dropouts to the failure to establish contact (through a reliable Internet connection). The possibility for teachers to learn about the students’ circumstances at home appeared as a benefit as it could reportedly lead to more effective practice at home (correct chair height, etc.).

***Factors related to the success*** of online education formed the fourth main code. In the analysis of advantages (37 codes) and disadvantages (175 codes), three shared subcodes were formed, namely about the *overall experience, the rate of progress, and the impact on students*. We first present the results for the solely negative subcodes. In the subcategory of cancelled events, we included all events cancelled or rescheduled as a result of the pandemic, such as concerts, diploma concerts, or competitions, and also considered the loss of routine in terms of concert performance. Among the subcodes, an important group related to the impact of external factors such as the occasional unfeasibility of uninterrupted learning (e.g. a sibling or parent interfering with the class), a decline in quality, or superficial work.

The general experience reflected a number of difficulties. A sizable majority of respondents questioned whether the online teaching of music, which they considered to be a temporary inadequate solution, was successfully implemented. It was not deemed feasible to teach beginners in this way. However, many respondents felt positive about the attitude and interest displayed by their students. The experiences on the extent of progress were also mixed. Some felt that the work was effective, while others questioned the effectiveness and efficiency of online music education, saying that it slowed progress and sometimes even led to dropouts. There were also divergent responses regarding the impact on students. Positive experiences included students’ preference to learn in their home environment and the positive impact of online education on the progress of students with learning disabilities. Those who expressed negative views mentioned the conservation of mistakes, students with lower abilities falling behind, and disadvantaged students dropping out. Finally, as a positive result, several respondents reported that they intended to use online education as a complementary teaching method in the future.

There was also ambiguity in the evaluation of **factors related to *mental and physical wellbeing*** (69 negative codes, 36 positive codes). This is illustrated by the fact that motivation emerged as a key element from both aspects: while some reported a decrease in motivation (both among teachers and students), others experienced an increase in motivation due to the new stimuli. However, the overall impact of online education on mental health is apparent from the frequent mentions of words with negative connotations such as tiring, stressful, depressive symptoms, feelings of isolation, increased stress, seclusion, and a general lack of enjoyment of online education. Despite the difficulties, it is an encouraging result that in general many children felt that they were not overburdened at home and were therefore fresher, more relaxed, and more balanced than usual.

While ambiguity was observed as regards mental factors, physical effects were evaluated only negatively. The most common responses mentioned the inconvenience of sitting in front of a computer all the time, which was considered to be exhausting, to impair and drain vision, to damage the eyes, and to cause back pain. Some respondents also complained of insomnia.

There was a marked diversity in the evaluation of ***spatial and temporal factors***, but while for the other main codes a clear predominance of negative aspects was observed, in this case positive thoughts (105 codes) outweighed negative ones (24 codes). In creating the subcodes, we initially aimed to separate spatial and temporal phenomena, but the complexity of the responses prompted us to create an intermediate subcode in which the two dimensions appeared in close association.

The responses showed a clearly positive assessment of purely *temporal factors* (68 codes). The possibility of flexible scheduling was identified as a significant advantage of online education for both teachers and students. Students had more time to practise and, as highlighted by some, had more freedom to work through the material they had to learn, allowing them to follow their individual pace of development. Besides the positive responses, a few teachers perceived temporal factors as a clear disadvantage. Some felt that online education was more time-consuming than traditional teaching as it required more preparation, thus increasing the number of hours spent working.

As mentioned, we also created an intermediate subcode for answers which related to both space and time. The temporal and spatial factors were also dominated by positive experiences (25 codes). Most respondents highlighted the fact that not having to commute allowed them to be at home more and to spend more time with their family. In contrast, there were some who felt that because of this the boundary between work and private life faded.

The experience of strictly *spatial factors* (12 codes) could only be considered as a fraction of the size of the main code. The convenience of working from home made this form of teaching particularly advantageous for teachers who normally commuted from far away. Online teaching was often disruptive to family or neighbours, and teaching was made more difficult by the inability to send the score to the student. The intimacy of music teaching was demonstrated by respondents who expressed that they missed the space in which they had worked.

The main code for factors related to preventive measures against the virus, and thus to ***health protection***, was the only one to feature positive responses only (9 codes). However, contrary to our expectations, very few respondents included in their answers the prevention of viral diseases as a benefit of online education.

As indicated above, we observed the interesting phenomenon that the responses on benefits often included negative comments (118 codes). This suggests that the negative feelings associated with online music education were so strong that teachers could only think of negative aspects even when asked about potential benefits. Several respondents felt that online music education had no advantages (71 codes), with face-to-face lessons deemed much more effective. In addition, various other opinions partially also featured disadvantages. According to these views, online teaching was insufficient, cumbersome, and less effective in the long term, poor sound quality was common, and the overall technical background was not good enough. In particular, a number of respondents felt that this form of teaching was only sufficient not to lose progress, as a supplement (10 codes). These opinions, while negative, may also allow for optimism as they implicitly relate to the potential usefulness of online education. Surprisingly, the opposite of this phenomenon was also observed, with some positive experiences listed among the disadvantages (10 codes). There was very little in common in these responses, which focussed on small, isolated segments. For example, it was possible to make good progress in music theory or music history, older students used digital tools confidently, and children with above-average ability displayed progress. It should also be pointed out that there were some who felt that online education could be a good way to learn the basics and that it had advantages as a supplementary form of education.

**Fig. 2:**
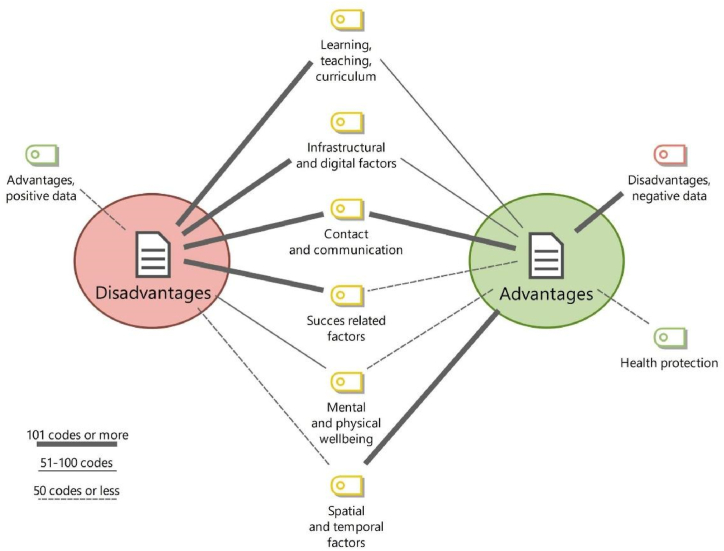
Visual map of the links between advantages and disadvantages with main codes. Source: OME Database 2021.

Fig. 2 summarises the strength of the links between advantages and disadvantages and across main codes. We distinguished three potential levels of the strength of the link, depending on the frequency of codes. It is striking that only the factors related to contact and communication showed a strong link with both negative and positive opinions. Moreover, both disadvantages and advantages were partially recorded under the opposite evaluation, but while the number of advantages recorded among disadvantages was negligible, negative aspects under main code for advantages were significant in number.

## 5 Summary

The content analysis and the frequency of codes revealed that most responses were based on negative experiences. “*As experiences, surveys, and studies have shown, effective art education is based on personal presence*” [27], so it is not a surprising result that most respondents considered the lack of personal contact as a major problem. The majority of respondents experienced the digital environment of online education negatively. Although respondents identified flexible scheduling and the time saved on commuting as significant advantages, the greatest benefit of online music education was that it allowed lessons to be carried out at all, albeit with some loss of efficiency. A somewhat surprising result is that the aspect of health protection was not given as high a priority as hypothesised, with the main code for this aspect ranked last by frequency.

Our research clearly showed that various innovations of online education, including the flexibility of scheduling and lessons, could be applied to in-person education in the future. Individual learning enhanced several students’ competences in self-regulation and autonomy. Using a virtual space made education independent of location, which led to time and economic savings by avoiding travel. Online classes also allowed the participation of students with disabilities or illnesses.

In conclusion, certain elements of online music education can be implemented in practice in the future. This would be useful and even necessary to study comprehensively.

## 6 Limitations

Some limitations should be considered in the current study. First, the study has just one data collection point, used self-reporting questionnaires. As a result, the sample size is limited. Second, the convenience sample from our study cannot be considered representative of Hungary, which limits the generalizability of the results. Finally, in order to obtain more precise and deeper information about this research topic, it is necessary to supplement the research with an interview method in the future.

## Institutional review board statement

The study was conducted according to the guidelines of the Declaration of Helsinki, and approved by the Institutional Review Board of Doctoral Program on Educational Sciences, University of Debrecen, Hungary (5/2020).

## Data availability statement

The data sets generated during and/or analyzed during the current study are available from the corresponding author on reasonable request.

## CRediT authorship contribution statement

**Judit Váradi:** Writing – review & editing, Writing – original draft, Validation, Resources, Project administration, Methodology, Funding acquisition, Formal analysis, Data curation, Conceptualization. **J. Miklós Radócz:** Writing – review & editing, Writing – original draft, Methodology, Formal analysis. **Ádám Mike:** Writing – review & editing, Writing – original draft, Methodology. **Zoltán Óváry:** Writing – original draft. **Gabriella Józsa:** Writing – review & editing, Writing – original draft, Methodology, Conceptualization.

## Declaration of competing interest

The authors declare that they have no known competing financial interests or personal relationships that could have appeared to influence the work reported in this paper.
