# Supplementary material for: Lessons from the COVID pandemic in music education the advantages and disadvantages of online music education
Source: Heliyon. 2024 Aug 2;10(15):e35357. doi: 10.1016/j.heliyon.2024.e35357 (PMC11336562; doi:10.1016/j.heliyon.2024.e35357)
Supplement: Multimedia component 1 [file mmc1.docx]

QUESTIONNAIRE FOR PRIMARY SCHOOL MUSIC TEACHERS

Dear Colleague!

The Music Pedagogy Research Group of the Doctoral Program in Education and Cultural Studies of the Doctoral School of Human Sciences of the University of Debrecen is conducting a survey on the experiences of online art education at primary level, commissioned and supported by the Foundation for the Art of the Future and Hungarian Academy of Arts' Research Institute for Art Theory and Methodology.

THE RESPONSE IS VOLUNTARY!

By completing it, you would greatly help us in our work.

Your answers will be treated confidentially and completely IN SILENCE. You can enter your answers by underlining, circling, or typing your answer in the appropriate spaces.

DEMOGRAPHIC ISSUES

1. Your age is ………. (please write with a number)

2. Gender a) Female b) Male

3. What type of settlement do you teach in?

a) capital city

b) county seat, city with county rights

c) city 4) village - over 5,000 people

d) village - less than 5 thousand people

4. Please write the county where you teach.

…………………………………………………………………………………

5. How many years have you been teaching? ……… year

6. The nature of your employment:

a) full-time b) part-time c) hourly rate d) other, namely………………………

7. Please indicate your job title: (More than one answer may be indicated.)

a) director

b) deputy director

d) work community leader

e) music teacher

f) other, namely………………………………..

8. What degree do you have? (If you have more than one, please list them all.)

1^st^ degree……………………………………………………………………………………… 2^nd^ degree……………………………………………………………………………………… 3^rd^ degree………………………………………………………………………………...

9. Are you currently participating in any professional training?

a) yes

b) no

c) If yes, then what? ………………………………………………………………………….

10. How many years have you been working as a teacher? ……(please write with a number)

11. How many years have you worked at this school? ………… (please write with a number)

12. What is your degree?

a) Trainee

b) Teacher I.

c) Teacher II.

d) Master teacher

e). Research teacher

13. What subject(s) and instrument(s) did you teach in the 2019/2020 school year?

a). …………………………………

b). …………………………………

c)….……………………………….

14. What subject(s) and instrument(s) did you teach in the 2020/2021 school year?

a). …………………………………

b). …………………………………

c)….……………………………….

15. Have you participated in any professional program or conference in the last 3 years?

a) yes, namely..……………………………………………………………………………….

b). no. What is the reason?……………...…………………………………………………….

ONLINE EDUCATION

16. In addition to your teacher degree, have you participated in any training that contributes to the development of your digital competence?

a) yes

b) no

17. Before online education, did you use digital tools in your work?

a) yes

b) no

18. Did you use your own devices for online education?

a) yes

b) no

19. Has the school provided any tools for digital education?

a) yes

b) no

20. Which of the listed devices did you use during the online education?

(More than one answer can be checked.)

a) desktop computer

b) laptop/notebook

c) tablets

d) smartphone

e) e-book

f) I don't have my own device

g) other, namely……………

21. During the period of extraordinary digital education, what percentage of your students were you able to reach during online classes?

a) I didn't reach anyone.

b) 1-20%

c) 21-40%

d) 41-50%

e) 51-70%

f) 71-80%

g) 81-100%

22. Which Internet platform was used for teaching?

(More than one answer can be checked)

a) Microsoft Teams

b) Chalk

c) Moodle

d) Facebook

e) Instagram

f) EduBase

g) Kahoot

h) Redmenta

i) Youtube

j) email

k) Skype

l) Webex

m) Discord

n) Zoom

o) Neo lms

p) Google Classroom

q) Messenger

r) Google Meets

s) Google Hangouts

t) MaxWhere

u) Google Drive

v) Other,…………………………………..

23. Has the school provided an internet interface for online teaching?

a) yes

b) no

24. How would you rate your own level of technological knowledge in the period BEFORE digital education?

minimal 1 2 3 4 5 excellent

25. How would you rate your current level of technological knowledge?

minimal 1 2 3 4 5 excellent

26. Did you create your own curriculum during the digital transition?

a) yes

b) no

27.1. If you answered yes to the previous question, what COURSE was it and on which internet interface? ……………………………………………………………………………

28. In general, to what extent do you agree with the following statements about digital arts education?

(1 strongly disagree -2 strongly disagree -3 strongly agree -4 strongly agree -5-strongly agree)

a) It takes more time to prepare for classes.

b) The lack of personal contact bothers me.

c) Students can be motivated well.

d) My students had instruments at home.

e) They could create a relaxed environment for the student.

f) Digital education forced me to do extra administration.

g) I was able to give learners a sense of achievement.

h) I could organise a concert with my students online.

i) I gave students live online lessons.

j) The student sent me recordings instead of lessons.

k) Teaching is more effective online.

l) my students practiced more during the emergency.

m) Students in my class have interrupted their studies as a result of online teaching.

n) Fewer students want to learn music after online education.

o) The number of students in my class has not decreased since September.

p) I am a member of an online professional group.

q) My work has been helped by online professional groups.

r) I have consulted my own colleagues about problems that arise.

s) I have asked for help with problems that arise in social media professional groups.

t) Students have met the expected requirements.

Thank you for helping our work by filling out the questionnaire! The research was carried out with the support of the Institute of Art Theory and Methodology of the Hungarian Academy of Arts.
